# Febrile infection-related Epilepsy Syndrome (FIRES): a severe encephalopathy with status epilepticus. Literature review and presentation of two new cases

**DOI:** 10.1186/s13052-022-01389-1

**Published:** 2022-12-16

**Authors:** Piero Pavone, Giovanni Corsello, Umberto Raucci, Riccardo Lubrano, Enrico Parano, Martino Ruggieri, Filippo Greco, Silvia Marino, Raffaele Falsaperla

**Affiliations:** 1 Department of Clinical and Experimental Medicine, University Hospital “Policlinico-San Marco”, Catania Catania, Italy; 2 Department of Health Promotion, Mather and Child Care, Internal Medicine and Medical Specialities, University of Palermo, Palermo, Italy; 3 Department of Emergency and Clinical Pediatrics, Bambin Gesù Children’s Hospital IRCCS, Rome, Italy; 4 Pediatrics and Neonatology Unit, Maternal-Child Department, Santa Maria Goretti Hospital, Sapienza University of Rome, Latina, Italy; 5 Unit of Catania, Institute for Research and Biomedical Innovation (IRIB), National Council of Research, Catania, Italy; 6 Unit of Pediatrics and Pediatric Emergency, AOU “Policlinico”- PO “San Marco”, University of Catania, Catania, Italy

**Keywords:** FIRES, Encephalopathy, NORSE, Status epilepticus

## Abstract

FIRES is defined as a disorder that requires a prior febrile infection starting between 2 weeks and 24 h before the onset of the refractory status epilepticus with or without fever at the onset of status epilepticus. The patients, previously normal, present in the acute phase recurrent seizures and status epilepticus followed by a severe course with usually persistent seizures and residual cognitive impairment. Boundary with “new onset refractory status epilepticus (NORSE) has not clearly established. Pathogenetic hypothesis includes inflammatory or autoimmune mechanism with a possible genetic predisposition for an immune response dysfunction.

Various types of treatment have been proposed for the treatment of the acute phase of the disorder to block the rapid seizures evolution to status epilepticus and to treat status epilepticus itself. Prognosis is usually severe both for control of the seizures and for cognitive involvement.

FIRES is an uncommon but severe disorder which must be carefully considered in the differential diagnosis with other epileptic encephalopathy.

## Introduction

Status Epilepticus (SE) of inflammatory origin recognizes 2 main etiologic events: the infectious, and the autoimmune cause: a third category is represented by disorders in which the inflammation event is suspected but still now not clearly demonstrated. The SE of infective origin include classic viral, bacterial, parasitic meningoencephalitis, acute abscesses, empyema, or prior disease. [1]. The group of Autoimmune Encephalitis (AE) manifesting with SE includes several disorders mediated by antibodies as those acting against the gamma-aminobutyric acid receptor (GABA R) A [2, 3], and (GABA R) B [4], leucine-rich glioma inactivated 1 (LGI1) [5], and with less frequency disorders of antibodies mediated against anti-N-methyl-D-aspartate receptor (NMDAR) [6], alfa-amino-3-hydroxy-5-methyl-4-isoxazoleproprionic acid receptor (AMPAR), [7] contactin-associated protein-2 receptor (CASPR 2), [8] and several other antibodies mediated disorders. [2]. An autoimmune pathogenetic mechanism has also been suggested but not confirmed in other severe encephalopathies presenting with SE as the Rasmussen’s Encephalitis (RE), New-Onset Status Epilepticus (NORSE) and Febrile Infection-Related Epilepsy syndrome (FIRES). 

Rasmussen’s Encephalitis (RE) is a very rare, chronic inflammatory neurological disease that usually affects only one hemisphere of the brain. Affected patients, previously healthy, manifest frequent, motor, unilateral, refractories seizures and status epilepticus, associated with progressive hemiparesis, cognitive decline, and unilateral hemispheric atrophy. Immune event has been thought to have a possible role in the pathogenesis of RE [9, 10] and alpha7 nAChR antibodies were reported by Watson et al. [11] in two of nine patients affected by RE. In RE, other pathogenetic mechanisms were also hypothesized including cytotoxic T-cell on the basis of the brain CD8 + T cell infiltration [12]. Wang et al. [13] compared Herpes Simplex Virus (HHVs) infection and relevant immune response among brain tissues from RE, temporal lobe epilepsy (TLE), and traumatic brain injury (TBI): they found, in comparison with TBI, both RE and TLE to have prevalent HHV infection and immune response in brain tissue. NORSE has been recently defined as a clinical presentation, and not a specific diagnosis: the patient without active epilepsy or other preexisting relevant neurological disorders, including absence of a previously clear acute or active structural, toxic or metabolic cause, manifest with new onset Refractory Status Epilepticus (RSE). FIRES is considered a subgroup of NORSE, in which a febrile infection starting between 2 weeks and 24 h precedes the onset of RSE in individuals without a previous active epilepsy, and without a clear acute or active structural, toxic or metabolic cause. [14–16]

An update on pathogenesis, clinical features, modality of treatment and outcome in patients affected by FIRES are herewith discussed with clinical description of 2 new cases.

### Clinical reports

#### Case 1

A 7 year-old boy came to the Pediatric Department University of Catania, Italy for recurrent episodes of focal seizures of short duration started during the night. At admission, the parents referred that the child eight days before the admission complained from a febrile episode lasted two days with mild temperature and treated with paracetamol. Family history was irrelevant for specific neurologic disorders and in the child previous neurologic manifestations were denied. He is the first child and the older sister is healthy. At the conception the mother was 27 years old and the father 29. The mother denied to have had infectious diseases and to have used drugs or alcohol during the gestation. Fetal movements were regularly felt and fetal ultrasound examination was normal. The child was born at term by spontaneous delivery with birth weight of 2.800 gr, height 49 cm and head circumference of 35 cm (all within normal range). Neonatal period was uneventful and developmental steps reached regularly for the age. The parents complained that the child suffered by frequent airway infections. Vaccinations were regularly conducted. He attended primary school with good results. Soon after the admission the child was submitted to treatment with i.v. midazolam (0.4 mg/Kg/hours) in a tempt to stop the seizures, but the seizures were persistent and more frequent in spite of the repeated i.v. midazolam treatment. At physical examination no malformation anomalies were observed. The ear, heart, respiratory tract, and abdominal organs were normal. Upon the neurologic examination the child was pained with feeble voice in the interval between the seizures. Patellar reflexes were particularly expressed. Fundus examination was normal. Hematologic findings including blood count, electrolytes, plasma and urine amino acids, thyroid testing, organic acids, plasma purine, and total cholesterol were normal. Hematologic results for viral infections were negative. The EEG recording showed the presence of spike and waves particularly evident in the fronto-temporal regions. The Brain MRI was not indicative. Cerebrospinal fluid (CSF) showed normal color, number of cells, glucose, proteins and albumin. CSF antibodies against GABA A, GABA B, LGI1, and NMDAR were negative and CSF polymerase chain reaction analyses were negative for viral agents. A new EEG showed diffuse delta-theta background. At the beginning of the fourth day as the seizures continued to be present with major frequency and duration a diagnosis of epileptic encephalopathy with convulsive status epilepticus was made and the child was transferred in the section of Pediatric Intensive Care Unit (PICU) where he was intubated and treated with barbiturate induced comae. After 4 days, the child was extubated and readmitted to the Pediatric Department. Antiepileptic treatment was started with topiramate (5 mg/Kg/day) and sodium valproate (20 mg/Kg/day). The child was discharged with this treatment. During the course of 2 years, a progressive reduction of the frequency of the seizures was reported, but seizures were still present (1–2 at month). The child follows sittings of neuropsychiatric rehabilitation therapy. He attends school with poor profit. He shows a moderate/severe cognitive delay with I.Q. of 38 at WISC-IV.

#### Case 2

A 10 year-old boy came to the Pediatric Department University of Catania, Italy for recurrent episode of focal seizures of short duration started two days before admission. The parents referred that the seizures appeared suddenly and that the child was grown normally up without specific clinical problems. They also referred that 12 days before the admission the child complained from a febrile episode involving the upper respiratory airway with high temperature for three days of duration and treated with paracetamol. Family history is negative for neurologic disorders. The child is second born and an older brother 18 year-old is healthy. The age of the mother at the conception was 36 years and 38 the age of the father. The gestation was conducted normally without infectious disorders. Fetal movements were regularly felt and no anomalies were found at the fetal ultrasound. The child was born at term by spontaneous delivery with weight, height and head circumference within the normal limits. Vaccinations were regularly conducted. The developmental steps were regularly reached and the child attended secondary school regularly with normal somatic growth parameter. The convulsive episodes firstly passed unnoticed by the parents. At admission the seizures were focal with secondary generalization and were treated with i.v. midazolam (0.4 mg/Kg/hours) and repeated at the onset of the recurrent episodes. At physical examination no malformation anomalies were observed and as well heart, respiratory tract were normal. Liver and spleen were within normal limits. The neurologic examination showed a pained child, with clouded sensorium and feeble voice at the interval between the seizures. These were intermittent with seizures of motor type with frequent recurrence and short duration. Routine hematologic findings plasma and urine amino acids, thyroid testing, organic acids, plasma purine, and total cholesterol were normal as well hematologic tests for viral infections. Fundus oculi was normal. No anomalies were found at the Brain MRI. Otoacoustic emissions test was normal. The EEG recording showed slowing background and presence of multifocal spike and waves particularly evident in the fronto-temporal regions. Color, numbers of cells, glucose, proteins, and albumin were normal and no antibodies against GABA A, GABA B, LGI1, and NMDAR were reported at the CSF examination. CSF polymerase chain reaction analyses were negative for viral agents. Anti-seizures treatments was started with topiramate (5 mg/Kg/day) and with sodium valproate (20 mg/Kg/die). After five days as the clinical condition seriously worsened with onset of convulsive status epilepticus the child was transferred at the PICU section where he was intubated and submitted to barbiturate induced coma. He was discharged by PICU after three days with anticonvulsant drug therapy consisting of topiramate (5 mg/Kg/day), sodium valproate (20 mg/Kg/day) and levetiracetam (30/mg/Kg/day). In the five years follow up the child showed a gradual reduction of the frequency of seizures presently 1–2 at month, with cognitive involvement (I.Q. 36 at WISC-IV) and poor scholastic performance. He follows sessions of neuropsychiatric rehabilitation therapy.

## Methods

The literature review was conducted by collecting clinical trials, primary research, and reviews from online bibliographic databases (MEDLINE, Embase, PubMed, Cochrane Central, and scopus, selected from 2000 to January 2022. The key search derived from the medical subject heading terms were pertaining to Autoimmune Encephalitis, New-Onset Status Epilepticus (NORSE), Fever Induced Refractory Epileptic syndromes, Febrile Infection-Related Epilepsy Syndrome (FIRES), Rasmussen ‘s encephalitis. Relevant studies were manually examined and included in the present reference list. After removing duplicate records, the main search results were included.

## Discussion

Both the probands here presented, showed the classical clinical manifestations of the FIRES and typical course with sequelae of moderate-severe cognitive impairment and persistence of seizures. Many clinical aspects of patients affected by the FIRES including pathogenesis, clinical manifestations with distinctive phases, way at diagnosis, different options of treatment, and outcome deserves to be discussed.

### FIRES

FIRES is a disorder which manifests with previous febrile episode and subsequent epileptic encephalopathy affecting mainly the pediatric age with a severe, usually striking neurological outcome. The term was coined by van Baalen et al. [17] in his article published in 2010. Similar clinical manifestations of FIRES were previously reported and termed as “acute encephalitis with refractory, repetitive partial seizures” and as “fever-induced refractory epileptic encephalopathy in school age children”. FIRES is an uncommon disorder, but incidence may be underestimated as the differential diagnosis with other types of SE of inflammatory origin is not easy to distinguish. Annual incidence and prevalence of FIRES in children and adolescents has been estimated by van Baalen et al. [18] on 1/ 1.000.000 and 1/ 100.000, respectively.

### Pathogenesis

Pathogenesis of FIRES is not completely clarified and there are no evidence of the factors involved in this disorder. Presently at the basis of the disorder, the most accredited hypothesis is an inflammatory or autoimmune mechanism with a possible genetic predisposition for anomalous immune response. Indeed, familial incidence of FIRES has not been reported and genetic studies are lacking to supply sufficient results. In FIRES patients, genes associated with fever-sensitive epilepsy as sodium voltage-gated channel protein type 1 subunit alpha (*SCN1A*), DNA polymerase subunit gamma- 1 (*POLG*) and protocaldherin-19 (*PCDH19*) were analyzed and no mutations were reported [19] as well no mutations were found in genes associated with infection-triggered encephalopathy and SE. [20]. Inflammatory mechanism in FIRES is the most accredited pathogenetic event. This hypothesis has been supported by the frequent observation of increased (usually mild) number of leucocytes in the spinal liquor and also by the high levels of proinflammatory cytokines/chemokines reported in serum and liquor in FIRES patients. [21, 22]. Progressive accumulation of the cascade inflammatory products may be cause of the lowering of the epileptic threshold with onset of seizures as supposed by Vezzani and Viviani [23], To further support the role of inflammation on pathogenesis of FIRES there is a report of Saitoh et al. [24] performed in 19 Japanese patients who report on genotyped polymorphisms frequency of the IL1B, IL6, IL10, Tumor necrosis factor alfa (TNFA), interleukin 1 receptor antagonist (IL1RN), SCN1A, and SCN2A genes in FIRES patients compared with case-controls. Concerning the IL1RN, the authors [24] found the frequency of a variable number of tandem repeat (VNTR) allele, RN2, significantly higher in the FIRES patients than in control (*p* = 0.0067), and A allele at rs4251981 in 5’ upstream of IL1RN with borderline significance (*p* = 0.015). Haplotype containing RN2 has been found to be with an increased risk of FIRES (OR 3.88, 95% CI.1.40–10.8, *p* = 0.057). The authors underline that IL1RN VNTR RN2 allele frequency was significantly high in FIRES patients. The report of the association between FIRES and an IL1RN gene polymorphism supports the hypothesis of the inflammation mediated pathogenesis. [25, 26] Moreover, the biphasic clinical course of the syndrome is suggestive of an immune disorder produced by an infection event even if the worsening of the clinical course seems not to be directly correlated. [25]. In a study of Hsieh et al. (27) the evaluation of TLR1-9 responses (IL-6, IL-8, IL-12p40, INF-*alfa,* INF gamma, and TNF-alfa) in peripheral blood mononuclear cells (PBMCs) and monocyte-derived dendritic cells (MDDCs) were compared with those of children with febrile seizures and non-refractory epilepsy with/without underlying encephalitis /encephalopathy: the authors [27] have found a Toll-like receptor3 (TLR3), TLR4,TLR7/8 and TLR9 responses impaired probably caused to their weakened phagocytosis and decreased T regulatory cells. Cytokine pathway, specifically mediated through IL-1Beta has been involved in both human and animals models of epilepsy and FIRES [16]. Dysregulation of the balance of IL-1Beta activation and inhibition may act on the onset of epilepsy. [16]

### Clinical manifestations and distinctive phases

The age of presentation of the disorder ranges in children between 7 and 10 years of age with a prevalence of the male gender and a psychomotor development previously normal. The clinical manifestations of FIRES tend to appear following three distinctive phases [28]. The first phase is characterized by a banal febrile episode with usually no high temperature, cough and/or diarrhea of short days duration such do not cause familial apprehension. The second phase starts after a period of wellbeing lasting between 24 h and 2 weeks and presenting with an acute, sudden, recurrent episodes of afebrile focal seizures (in some case with secondary generalization) of short duration, and interposed by confusional state and poor reactivity. The seizures barely respond to anticonvulsant treatment and tend to rapidly evolve into Refractory Status Epilepticus (RSE). According to the severity of the disorder the period of RSE may last for a few days to weeks. The third phase, the chronic period is usually manifested by neurological sequelae with more or less cognitive and language impairment, motor dysfunction and drugs resistant seizures.

Both the probands here reported have covered the three typical phases of the FIRES with a previous mild febrile episodes followed a few days after by recurrent focal seizure with rapidly developed RSE which has required treatment in PICU and subsequent neurological dysfunction.

### Diagnosis

Diagnosis of FIRES, in the first phase of the disorder, is not feasible as currently no specific test are available [28]. On the second stage of the disorder with the acute onset of afebrile seizures some diagnostic indication can arise analyzing the clinical and laboratory results of the subject with previous negative personal history of seizures and with no specific hematological and liquor laboratory anomalies. The rapid evolution of the seizures to RSE are the critical points for a correct diagnosis of FIRES according to the results of the clinical examination, hematologic test, serial electroencephalogram recording, and not specific anomalies at brain MRI, and at the liquor examination. Neurologic examination between the seizures shows signs of confusional state and poor reaction, whereas cranial nerves are not involved and meningeal signs are absent. The remaining of clinical examination is normal including levels of spleen and liver. Blood tests are unrevealing. As reported by Caraballo et al. [29] the seizures, in the acute phase are focal with possible secondary generalization, in association sometime to autonomic manifestations such as pallor, apnea, and cyanosis. These authors [29] in 12 patients with FIRES at interictal EEG recordings report on a diffuse delta-theta background slowing and at the initial EEG recordings a temporal seizure onset in four children, fronto-temporal in four, frontal in two, and fronto-parietal in two, respectively. All the 12 patients rapidly developed multifocal and independent seizures [29]. In FIRES, Rachfalska et al. [30] refer that seizures are usually focal or secondarily generalized without regaining consciousness between them. Multifocal seizures are present in most of the patients and in some of them, the foci may migrate indicating a compromision of both the hemispheres. The EEG initially shows high-voltage slow background activity with subsequent development of interictal epileptiform discharges with variable spatial distribution. Ictal recording are most commonly located in temporal, fronto-temporal and frontal regions including perisylvian area [30]. Wu et al. [31] collected 92 patients with NORSE among which 90% were affected by FIRES. Interictal EEG recording showed slow wave, suppression, burst suppression, and multifocal or focal discharges. In 72 patients, focal interictal discharges were found in 36.1% of cases, extensive or multifocal discharges in 61.1% and in 2.8% periodic discharges were registered. In the remaining 20 patients, no discharges were reported at the interictal period. During the ictal period, a rhythmic theta or beta wave; focal sharp or spike and slow wave; or generalized discharge were reported. Either sustained or intermittent, were present in the background. During the SE phase or after the SE interruption, epileptic EEG signs were noted in 48 patients. To note, in this series 21 patients had nonconvulsive status epilepticus presentation (NCSE) [31]. Farias-Moeller et al. [32], in a retrospective single-center study of seven children with FIRES, report the EEG recordings and distinguished the results obtained in two phases: the initial, with 12 h of recording and the subsequent with 12 h of recording performed prior to initiation of the medically induced burst suppression (BS). The EEG background for all the patients showed extreme delta brush (EDB) with paroxysmal beta-delta complex consisting of 15–18 Hz beta activity superimposed over 1–3 Hz delta in the frontal and central regions. On the subsequent EEG recording, the typical seizure burden was found higher at 1–5 seizures/h. A specific seizure pattern was reported in six out the seven patients consisting of focal activity > 10 Hz of small to moderate amplitude evolving to well-formed rhythmic spike and spike-wave complexes. In four of the patients, ictal activity shifted from one hemisphere to the contralateral. Most seizures in the first 12 h of recording came from frontal, central, and temporal areas on either hemisphere; whereas in the subsequent EEG recording the seizures were found to come from occipital and parietal areas [32]. Together with EEG recording, brain MRI is a relevant diagnostic exam and it should be promptly required to exclude structural abnormalities and to proceed in further assessments including the CSF examination. Culleton et al. [33] collected a total of 129 MRI brain scans reports from the literature carried up during the acute phase of FIRES. The authors report than in the initial phase of the disorder in most of the patients the MRI is inconclusive for FIRES diagnosis. They refer that at initial presentation the most common finding was a normal brain MRI in 79/129 (61,2%).Temporal lobe (including the mesial temporal lobe structures and hippocampi), signal abnormalities unilateral and bilateral were reported in 33 out 129 cases (25.5%); basal ganglia changes in 9 out 129 (6.9%); insular and periinsular changes in 7 out 129 (5.4%) as well as cortical edema either focal and diffuse was reported in 6/129 (4.6%). Signal abnormalities in the thalamus and in brainstem were reported in 2/129 cases (1.5%), respectively, and less frequently multifocal subcortical infarcts and cerebellar edema and hemorrhage. The same authors [33] report results of brain MRI imaging in children with FIRES during the chronic phase of the disease: the available data were 97/131 (74%). Among these, normal brain imaging was reported in 18/97 (18.5%). The most frequent abnormalities were a generalized brain atrophy including ventriculomegaly reported in 48 out of 97 (49.4%), cerebellar atrophy was specifically noted in 3 out 78 (3.1%), mesial temporal lobe hyperintense signal changes and/or atrophy in 24 out 97 (18.5%), ventricular white matter hyperintensities in 9 out 97 (9.2%) or deep or subcortical white matter changes in 2 out 97 (2.1%). Moreover, Insular and periinsular hyperintensities were reported in 3 out 97 (3.1%), deep grey matter and thalamic hyperintensities signal changes in 5 out 97 (5.3%) and cortical hyperintensities in 2 out 97 (2.1%). In general, brain MRI performed at distant is reported to show bilateral temporal atrophy and T2 hypersignal in almost half of cases [33]. Lee and Chi [34] collected 29 patients affected by FIRES: abnormal brain MRI findings, were reported in the initial phase of the disorder in 38% of the patients and in 87% at the follow up. Serial magnetic resonance imaging were carried out during the course of FIRES in 7 children aged 3 months to 9 years and performed from the initial presentation through 18 years of follow up. Results showed progressive evolution from normal imaging to severe cerebral atrophy. After six to twelve months, most of the children showed moderate to severe cerebral atrophy and by 1 year, also cerebellar atrophy was noted [35]. Laboratory analysis are usually normal. Spinal liquor may express an increased number of leucocytes observed in over 50% of patients [30, 35] as well levels of proinflammatory cytokines and chemokines [22].

In our probands the initial seizures were focal, recurrent and of short duration with subsequent develop of RSE happened after 4 days and 5 days, respectively. Initial EEG showed spike and waves in the fronto-temporal region in case 1 and multifocal spike and waves more evident in fronto-temporal regions in case 2. In the initial phase, in both Brain MRI was normal as well all the laboratory analysis including cerebrospinal fluid.

### Treatment

- **a** Treatment of children with FIRES is related to the phase of the disorder and to different therapeutic options used to control seizures and to stop the developing RSE and other consequences. Various therapeutic solutions have been advanced, but no one has reached a consistent general approval. The first phase in patients with FIRES does not cause any trouble and for the increased temperature the common use of antipyretic drugs are justified. At the beginning of the second phase of the disorder and the onset of seizures usually of type recurrent, treatment with classical i.v. benzodiazepines as midazolam, clonazepam, lorazepam and diazepam in association with standard anticonvulsant drugs, as oral levetiracetam, valproic acid, and lacosamide, or other options are used [28]. The subsequent onset of RSE requires the use of more consistent pharmacological procedures including barbiturate induced coma with infusion of anesthetic drugs as midazolam, thiopental and propofol or other therapeutic systems. Classical anticonvulsants single or in association with levetiracetam, valproic acid,and lacosamide is proposed for the chronic third phase together with a neuropsychological and neuropsychiatric recovery.
- **b** Alternative types of treatment in FIRES have been proposed. Ketogenic Diet (KD) was experienced by Nabbout et al. [36] in nine patients with FIRES who were refractory to conventional antiepileptic treatment: in seven of these patients treatment with KD was efficacious with seizure cessation the 4–6 days following the onset of the diet with recovering of motor functions within the following weeks. KD was effective in the treatment of the seizures within a few months. [36]. In one patient initially responder to KD early disruption of the treatment resulted in relapse of SE and severe outcome with the death of the patient. Among the group of 77 patients collected by Kramer et al. [37] affected by FIRES, seven were treated with ketogenic diet (KD): among these, two showed borderline and 1 mild cognitive dysfunction, 3 severe cognitive dysfunction or vegetative status and 1 had exitus. None of the many antiepileptic drugs (AED) as well treatment with KD was effective in the reduction or stopping the epileptic seizures. One patient who had more than 100 seizures a day showed with KD treatment an excellent clinical answer with total cessation of seizures within 2 days of KD onset. Singh et al. [38] report on two patients with FIRES who were treated with KD during the acute phase of the illness and both experiences resolution of SE. Treatment was maintained for several months with KD and other anticonvulsant drugs with good outcome [38]. The benefit effect of KD has been reported to be related to anti-inflammatory properties of this treatment as reported in studies in animals. [16, 39]. However in the 29 patients collected by Lee and Chi in seven (7/29, 24%) treated with KD no beneficial effects were reported. [34]. In the group of 15 children reported by Patil et al. [40] an attempt with KD treatment was carried out in two patients in the later phases of the illness and the clinical results was poor.
- **c** On the basis of a presumptive involvement of the immunomodulatory and anti-inflammatory system, other therapeutic assessments has been employed in the treatment of FIRES including high-dose i.v. steroids, IVIG, plasmapheresis and other agents known to efficaciously interfere in the human immune dysfunction. [16, 25] Kramer et al. [37] in the group of 77 FIRES patients distinguished the clinical outcome obtained according to the treatment performed with high-dose intravenous steroid and patients treated with IVIG, respectively. In FIRES patients treated with high-dose intravenous steroid the results of cognitive outcome were the following: out of 20 patients six were normal; four borderline; three moderate-mild, one severe, six in vegetative state. The same authors [37] in the group of twenty-seven subjects treated with IVIG obtained the following results in cognitive outcome: six normal; ten borderline; one moderate; two severe and eight borderline. At the follow up for epilepsy, as regards the group of patients treated with classical anticonvulsants therapy, and/or steroid, IVIG, KD, among 66 of the 68 survived, 63 of them continued to have epilepsy refractory to any type of treatment and seizures continued without interruption following the acute phase of FIRES or they appeared after a lag of a few weeks to 3 months. The Anakinra, a recombinant, modified version of the human interleukin 1 receptor antagonist protein, used to treat anti-inflammatory disorders has been employed as an immune modulator for FIRES patients. Kenney-Jung et al. [41] treated with Anakinra a child presenting super-refractory status epilepticus FIRES –related which was well tolerated and effective. CSF analysis in the subject revealed increased levels of proinflammatory cytokines before the treatment that normalized after the treatment [41]. Two male children with FIRES unresponsive to multiple anti-epileptic drugs, first-line immune-modulation, kD, and cannabidiol were treated with Anakinra and deep brain stimulation of the centromedian thalamic nuclei (CMN-DBS), the last to reduce generalized seizures. [42] CMN-DBS stopped generalized seizures in both the children, whereas Anakinra had a favorable effect only in one of the children. A study of an international retrospective cohort of 25 FIRES children treated with Anakinra was reported by Lai et al. [7]: among 15 children in whom frequency of seizures was available, 11 of them exhibited > 50% seizure reduction at 1 week of treatment onset. The median interval between seizure onset and Anakinra treatment was 19 days ( 12–30 days) in children with seizure reduction; 27 days (13.5–37.5 days) in children without seizure reduction. Early Anakinra initiation was associated with shorter duration of mechanical ventilation, ICU, and hospital length of stay. (7) Cannabidiol has been used as potential treatment in patients with FIRES. Gofshteyn et al. [43] report on 7 patients no responsive to antiepileptic drugs to whom treatment with cannabidiol was started. In 6 out 7 of them treatment resulted in improvement in frequency and duration of the seizures: an average of 4 antiepileptic drugs were weaned, 5 patients were ambulatory, 1 walked with assistance, and 4 were verbal. One of these patients died due to multiorgans failure. The benefic action of the Cannabidiol on seizures has been related to a decreasing glutamate and gamma-aminobutyric acid synaptic transmission in the brain. Reduction in excitatory neurotransmitter release may result in an increase of the seizure threshold [28, 44]. In a patient reported by Babi et al. [44] abuse of synthetic cannabinoid was cause of severe complications with episodes of acute psychosis and new-onset refractory status epilepticus. Immunomodulatory treatment using singly Tolicizumab, tacrolimus, rituximab, canakinumab have given no clear clinical benefit [16, 29, 45, 46].Two children aged 2 and 16 years, respectively affected by FIRES were treated with Infusion of continuous intravenous magnesium sulfate and serum magnesium concentrations achieved ranging from 2.1 to 5 mmol/L. [47]. Seizure reduction and cessation were reported in 1 of the patient whose serum magnesium concentration achieved more than 3.0 mmol/L with no significant adverse effects [47].
- **d** In most cases, the clinical course of the disorder is rapidly progressive with seizures unresponsive to any anticonvulsants treatment and rapidly progressive passage in a short time to RSE. In these cases, the second-line intervention with pharmacological coma become justifiable. The anesthetic agents more used are infusion of midazolam and barbiturates (pentobarbital, thiopental) and, propofol. Seizure recurrence is often reported [28]. Treatment with anesthetics agents, above all when prolonged has been related with a worse cognitive outcome and a more severe course of the disorder [17].

### Outcome

FIRES is a complex and severe disorder in which recurrent, unresponsive to treatment seizures are the most prevalent clinical signs. In most of the cases treatment is elusive and no general agreement has been reached. Moreover, in several cases the transition from focal (or focal to generalized seizures) to SE is so rapid that the action of any treatment becomes inconclusive and recurrence to second-line with pharmacological coma becomes appropriate. In association with the difficult control of the seizures, cognitive impairment is another relevant clinical problem related to this disorder. Only one-third of the surviving patients shows a normal or borderline cognitive results; one –third shows mild to moderate intellectual disability whereas one-third presented with intellectual disability severe or vegetative state [28]. Cognitive impairment has been related to the duration of burst suppression coma and to the younger age of onset. In other words more severe is the presentation, the delayed treatment and the rapid course of the disorder more poor is the prognosis as regards seizures control and cognitive impairment. In our probands pharmacological coma resulted in the interruption of recurrence of seizures. At follow up the seizures are moderately controlled and the cognitive impairment was moderate-severe allowing a relatively normal everyday life.

## Conclusions

FIRES is a quite rare and mysterious disorder in which different clinical aspects need to be clarify:as regards to the pathogenesis what is the role of the inflammatory or the auto immune mechanism; the relationship between FIRES and NORSE and their common boundaries; the best options for the treatment, to avoid severe clinical neurological outcome.

## Data Availability

“Not applicable”.

## Abbreviations

FIRES: Febrile Infectious-Related Epilepsy
NORSE: New Onset Status Epilepticus
SE: Status Epilepticus
AE: Autoimmune Encephalitis
GABA R: Gamma-aminobutyric acid receptor
LGI 1: Leucine-rich glioma inactivated 1
NMDAR: Anti-N-methyl-D- aspartate receptor
AMPAR: Alfa-amino-3-hydroxy-5-methyl-4-isoxazoleproprionic acid receptor
CASPR 2: Contactin-associated protein-2 receptor
RE: Rasmussen’s encephalitis
HSV: Herpes simplex virus
TLE: Temporal lobe epilepsy
RSE: Refractory status epilepticus
PICU: Pediatric intensive care unit
SCN1A: Sodium voltage-gated channel protein type 1 subunit alpha
POLG: DNA polymerase subunit gamma-1
PCDH19: Protocaldherin-19
TNFA: Tumor necrosis factor alfa
IL1RN: Interleukin 1 receptor antagonist
VNTR: Variable number of tandem repeat
TL3: Toll-like receptor 3
PBMC: Peripheral blood mononuclear cells
NCSE: Non convulsive status epilepticus
EDB: Extreme delta brush
AED: Antiepileptic drugs
KD: Ketogenic diet
CMN-DBS: Centromedian nuclei-deep brain stimulation

## References

[1] Spatola M, Novy J, Du Pasquier R, Dalmau J, Rossetti AO (2015). Status epilepticus of inflammatory etiology: a cohort study. Neurology.

[2] Spatola M, Dalmau J (2017). Seizures and risk of epilepsy in autoimmune and other inflammatory encephalitis. Curr Opin Neurol.

[3] Petit-Pedrol M, Armangue T, Peng X, Bataller L, Cellucci T, Davis R, McCracken L, Martinez-Hernandez E, Mason WP, Kruer MC, Ritacco DG, Grisold W, Meaney BF, Alcalá C, Sillevis-Smitt P, Titulaer MJ, Balice-Gordon R, Graus F, Dalmau J (2014). Encephalitis with refractory seizures, status epilepticus, and antibodies to the GABAA receptor: a case series, characterisation of the antigen, and analysis of the effects of antibodies. Lancet Neurol.

[4] Lancaster E, Lai M, Peng X, Hughes E, Constantinescu R, Raizer J, Friedman D, Skeen MB, Grisold W, Kimura A, Ohta K, Iizuka T, Guzman M, Graus F, Moss SJ, Balice-Gordon R, Dalmau J (2010). Antibodies to the GABA(B) receptor in limbic encephalitis with seizures: case series and characterisation of the antigen. Lancet Neurol.

[5] Mayasi Y, Takhtani D, Garg N (2014). Leucine-rich glioma-inactivated protein 1 antibody encephalitis. A case report. Neurol Neuroimmunol Neuroinflamm.

[6] Dalmau J, Gleichman AJ, Hughes EG, Rossi JE, Peng X, Lai M, Dessain SK, Rosenfeld MR, Balice-Gordon R, Lynch DR (2008). Anti-NMDA-receptor encephalitis: case series and analysis of the effects of antibodies. Lancet Neurol.

[7] Lai M, Hughes EG, Peng X, Zhou L, Gleichman AJ, Shu H, Matà S, Kremens D, Vitaliani R, Geschwind MD, Bataller L, Kalb RG, Davis R, Graus F, Lynch DR, Balice-Gordon R, Dalmau J (2009). AMPA receptor antibodies in limbic encephalitis alter synaptic receptor location. Ann Neurol.

[8] van Sonderen A, Ariño H, Petit-Pedrol M, Leypoldt F, Körtvélyessy P, Wandinger KP, Lancaster E, Wirtz PW, Schreurs MW, SillevisSmitt PA, Graus F, Dalmau J, Titulaer MJ (2016). The clinical spectrum of Caspr2 antibody-associated disease. Neurology.

[9] Freeman JM (2005). Rasmussen's syndrome: progressive autoimmune multi-focal encephalopathy. Pediatr Neurol.

[10] Samanci B, Tektürk P, Tüzün E, Erdağ E, Kınay D, Yapıcı Z, Baykan B (2016). Neuronal autoantibodies in patients with Rasmussen's encephalitis. Epileptic Disord.

[11] Watson R, Jepson JE, Bermudez I, Alexander S, Hart Y, McKnight K, Roubertie A, Fecto F, Valmier J, Sattelle DB, Beeson D, Vincent A, Lang B (2005). Alpha7-acetylcholine receptor antibodies in two patients with Rasmussen encephalitis. Neurology.

[12] Bien CG, Bauer J, Deckwerth TL, Wiendl H, Deckert M, Wiestler OD, Schramm J, Elger CE, Lassmann H (2002). Destruction of neurons by cytotoxic T cells: a new pathogenic mechanism in Rasmussen's encephalitis. Ann Neurol.

[13] Wang YS, Liu D, Wang X, Luo QL, Ding L, Fan DY, Cai QL, Tang CY, Yang W, Guan YG, Li TF, Wang PG, Luan GM, An J (2022). Rasmussen's encephalitis is characterized by relatively lower production of IFN-β and activated cytotoxic T cell upon herpes viruses infection. J Neuroinflammation.

[14] Pavone P, Pappalardo XG, Parano E, Falsaperla R, Marino SD, Fink JK, Ruggieri M (2022). Fever –associated seizures or epilepsy: an overview of old and recent literature acquisitions. Front Pediatr.

[15] Hirsch LJ, Gaspard N, van Baalen A, Nabbout R, Demeret S, Loddenkemper T, Navarro V, Specchio N, Lagae L, Rossetti AO, Hocker S, Gofton TE, Abend NS, Gilmore EJ, Hahn C, Khosravani H, Rosenow F, Trinka E (2018). Proposed consensus definitions for new-onset refractory status epilepticus (NORSE), febrile infection-related epilepsy syndrome (FIRES), and related conditions. Epilepsia.

[16] Koh S, Wirrell E, Vezzani A, Nabbout R, Muscal E, Kaliakatsos M, Wickström R, Riviello JJ, Brunklaus A, Payne E, Valentin A, Wells E, Carpenter JL, Lee K, Lai YC, Eschbach K, Press CA, Gorman M, Stredny CM, Roche W, Mangum T (2021). Proposal to optimize evaluation and treatment of Febrile infection-related epilepsy syndrome (FIRES): a report from FIRES workshop. Epilepsia Open.

[17] Van Baalen A, Hausler M, Boor R, Rohr A, Sperner J,Sperner J et al. Febrile-infection-related epilepsy syndrome (FIRES): A nonencephalitic encephalopathy in childhood. Epilepsis Vol 51, issue 7 / p. 1323–1328. https//doi.org/10.1111/j.1528-1167.2010.02535.x 10.1111/j.1528-1167.2010.02535.x 20345937

[18] van Baalen A, Vezzani A, Häusler M, Kluger G (2017). Febrile infection-related epilepsy syndrome: clinical review and hypotheses of epileptogenesis. Neuropediatrics.

[19] Appenzeller S, Helbig I, Stephani U, Häusler M, Kluger G, Bungeroth M, Müller S, Kuhlenbäumer G, van Baalen A (2012). Febrile infection-related epilepsy syndrome (FIRES) is not caused by SCN1A, POLG, PCDH19 mutations or rare copy number variations. Dev Med Child Neurol.

[20] Gaspard N, Hirsch LJ, Sculier C, Loddenkemper T, van Baalen A, Lancrenon J, Emmery M, Specchio N, Farias-Moeller R, Wong N, Nabbout R (2018). New-onset refractory status epilepticus (NORSE) and febrile infection-related epilepsy syndrome (FIRES): State of the art and perspectives. Epilepsia.

[21] Sakuma H, Tanuma N, Kuki I, Takahashi Y, Shiomi M, Hayashi M (2015). Intrathecal overproduction of proinflammatory cytokines and chemokines in febrile infection-related refractory status epilepticus. J Neurol Neurosurg Psychiatry.

[22] Kothur K, Bandodkar S, Wienholt L, Chu S, Pope A, Gill D, Dale RC (2019). Etiology is the key determinant of neuroinflammation in epilepsy: elevation of cerebrospinal fluid cytokines and chemokines in febrile infection-related epilepsy syndrome and febrile status epilepticus. Epilepsia.

[23] Vezzani A, Viviani B (2015). Neuromodulatory properties of inflammatory cytokines and their impact on neuronal excitability. Neuropharmacology.

[24] Saitoh M, Kobayashi K, Ohmori I, Tanaka Y, Tanaka K, Inoue T, Horino A, Ohmura K, Kumakura A, Takei Y, Hirabayashi S, Kajimoto M, Uchida T, Yamazaki S, Shiihara T, Kumagai T, Kasai M, Terashima H, Kubota M, Mizuguchi M (2016). Cytokine-related and sodium channel polymorphism as candidate predisposing factors for childhood encephalopathy FIRES/AERRPS. J Neurol Sci.

[25] Serino D, Santarone ME, Caputo D, Fusco L (2019). Febrile infection-related epilepsy syndrome (FIRES): prevalence, impact and management strategies. Neuropsychiatr Dis Treat.

[26] Shyu CS, Lee HF, Chi CS, Chen CH (2008). Acute encephalitis with refractory, repetitive partial seizures. Brain Dev.

[27] Hsieh MY, Lin JJ, Hsia SH, Huang JL, Yeh KW, Chang KW, Lee WI (2020). Diminished toll-like receptor response in febrile infection-related epilepsy syndrome (FIRES). Biomed J.

[28] Yun-Jin Lee Febrile Infection-Related Epilepsy Syndrome: Refractory Status Epilepticus and Management Strategies. Ann Child Neurol. 2020;28(1):8–15. DOI: 10.26815/acn.2019.00283 10.1111/dmcn.14553 32372459

[29] Caraballo RH, Reyes G, Avaria MF, Buompadre MC, Gonzalez M, Fortini S, Cersosimo R (2013). Febrile infection-related epilepsy syndrome: a study of 12 patients. Seizure.

[30] Rachfalska N, Pietruszewski J, Paprocka J (2021). Dramatic Course of Paediatric Cryptogenic Febrile Infection-Related Epilepsy Syndrome with Unusual Chronic Phase Presentation-A Case Report with Literature Study. Brain Sci.

[31] Wu J, Lan X, Yan L, Hu Y, Hong S, Jiang L, Chen J (2021). A retrospective study of 92 children with new-onset refractory status epilepticus. Epilepsy Behav.

[32] Farias-Moeller R, Bartolini L, Staso K, Schreiber JM, Carpenter JL (2017). Early ictal and interictal patterns in FIRES: The sparks before the blaze. Epilepsia.

[33] Culleton S, Talenti G, Kaliakatsos M, Pujar S, D'Arco F (2019). The spectrum of neuroimaging findings in febrile infection-related epilepsy syndrome (FIRES): A literature review. Epilepsia.

[34] Lee HF, Chi CS (2018). Febrile infection-related epilepsy syndrome (FIRES): therapeutic complications, long-term neurological and neuroimaging follow-up. Seizure.

[35] Rivas-Coppola MS, Shah N, Choudhri AF, Morgan R, Wheless JW (2016). Chronological evolution of magnetic resonance imaging findings in children with febrile infection-related epilepsy syndrome. Pediatr Neurol.

[36] Nabbout R, Mazzuca M, Hubert P, Peudennier S, Allaire C, Flurin V, Aberastury M, Silva W, Dulac O (2010). Efficacy of ketogenic diet in severe refractory status epilepticus initiating fever induced refractory epileptic encephalopathy in school age children (FIRES). Epilepsia.

[37] Kramer U, Chi CS, Lin KL, Specchio N, Sahin M, Olson H, Nabbout R, Kluger G, Lin JJ, van Baalen A (2011). Febrile infection-related epilepsy syndrome (FIRES): pathogenesis, treatment, and outcome: a multicenter study on 77 children. Epilepsia.

[38] Singh RK, Joshi SM, Potter DM, Leber SM, Carlson MD, Shellhaas RA (2014). Cognitive outcomes in febrile infection-related epilepsy syndrome treated with the ketogenic diet. Pediatrics.

[39] Dupuis N, Curatolo N, Benoist JF, Auvin S (2015). Ketogenic diet exhibits anti-inflammatory properties. Epilepsia.

[40] Patil SB, Roy AG, Vinayan KP (2016). Clinical profile and treatment outcome of febrile infection-related epilepsy syndrome in South Indian children. Ann Indian Acad Neurol.

[41] Kenney-Jung DL, Vezzani A, Kahoud RJ, LaFrance-Corey RG, Ho ML, Muskardin TW, Wirrell EC, Howe CL, Payne ET (2016). Febrile infection-related epilepsy syndrome treated with anakinra. Ann Neurol.

[42] Sa M, Singh R, Pujar S, D'Arco F, Desai N, Eltze C, Hughes E, Al Obaidi M, Eleftheriou D, Tisdall M, Selway R, Cross JH, Kaliakatsos M, Valentin A (2019). Centromedian thalamic nuclei deep brain stimulation and Anakinra treatment for FIRES - Two different outcomes. Eur J Paediatr Neurol.

[43] Gofshteyn JS, Wilfong A, Devinsky O, Bluvstein J, Charuta J, Ciliberto MA, Laux L, Marsh ED (2017). Cannabidiol as a potential treatment for febrile infection-related epilepsy syndrome (FIRES) in the acute and chronic phases. J Child Neurol.

[44] Babi MA, Robinson CP, Maciel CB. A spicy status: Synthetic cannabinoid (spice) use and new-onset refractory status epilepticus-A case report and review of the literature. SAGE Open Med Case Rep. 2017 Dec 5;5:2050313X17745206. doi: 10.1177/2050313X17745206. 10.1177/2050313X17745206 PMC5721953 29238581

[45] Howell KB, Katanyuwong K, Mackay MT, Bailey CA, Scheffer IE, Freeman JL, Berkovic SF, Harvey AS (2012). Long-term follow-up of febrile infection-related epilepsy syndrome. Epilepsia.

[46] Jun JS, Lee ST, Kim R, Chu K, Lee SK (2018). Tocilizumab treatment for new onset refractory status epilepticus. Ann Neurol.

[47] Tan  WW, Chan  DWS, Lee  JH, Thomas  T, Menon  AP, Chan  YH (2015). Use of magnesium sulfate infusion for the management of Febrile Illness-related Epilepsy Syndrome: a case series. Child Neurol Open.

